# Association of a major tobacco tax increase in California with increased smoking cessation

**DOI:** 10.1093/jnci/djaf121

**Published:** 2025-06-18

**Authors:** Shu-Hong Zhu, Christopher M Anderson, Yue-Lin Zhuang, Hai-Yen Sung, Anthony C Gamst

**Affiliations:** The Herbert Wertheim School of Public Health and Human Longevity Science, University of California San Diego, La Jolla, CA, United States; Moores Cancer Center, University of California San Diego, La Jolla, CA, United States; Moores Cancer Center, University of California San Diego, La Jolla, CA, United States; Moores Cancer Center, University of California San Diego, La Jolla, CA, United States; Institute of Health and Aging, University of California San Francisco, San Francisco, CA, United States; Computational and Applied Statistics Laboratory, San Diego Supercomputer Center, Department of Mathematics, University of California San Diego, La Jolla, CA, United States

## Abstract

**Background:**

In November 2016, California voters approved Proposition 56, increasing the cigarette tax by $2.00 per pack and nearly tripling spending on tobacco prevention. This study examined whether the initiative was associated with increased smoking cessation.

**Methods:**

States in the United States were categorized into 3 groups: California, 18 other states (including the District of Columbia) that raised taxes, and 32 states that did not raise taxes. Tax and price increases, tobacco prevention spending per capita, 3-month smoking cessation rates using data from the Behavioral Risk Factor Surveillance System (*N* = 443 054), and the proportion of daily smoking were compared for 2014-2016 and 2017-2019 for these groups.

**Results:**

California had the largest increases in cigarette price (30.8%) and tobacco prevention spending (271.9%), both adjusted for inflation. Other states that raised taxes experienced price increases of 6.3% on average. The 3-month smoking cessation rate in California increased from 11.5% in 2014-2016 to 14.2% in 2017-2019 (*P* = .005). Among other states that raised taxes in that timeframe, cessation rates did not change significantly, from 8.6% to 8.7% (*P* = .755). Among states that did not raise taxes, cessation rates declined significantly, from 9.5% to 9.0% (*P* = .026). California also had a significant reduction in the proportion of daily smokers among those who did not quit (from 60.4% to 56.4%, *P* = .012).

**Conclusions:**

A major cigarette tax increase was associated with increased smoking cessation in California. Policies increasing tobacco taxes and re-investing new revenue in tobacco prevention can increase population cessation.

## Introduction

The prevalence of adult cigarette smoking in the United States declined dramatically from 42.4% in 1965 to 11.5% in 2021,^1^^,^^2^ but the number who smoke is still large—an estimated 28.3 million adults in 2021.^2^ Smoking is the leading cause of preventable disease, disability, and premature death in the United States, including about 30% of all cancer deaths.^3^^,^^4^ The United States government has developed a framework to support and accelerate smoking cessation as part of a larger goal to reduce cancer deaths by half within 25 years.^4^ This ambitious goal requires an all-hands-on-deck response, with governments at all levels doing everything they can to assist the effort, including enacting smoke-free policies, conducting anti-smoking media campaigns, restricting tobacco marketing, and raising tobacco excise taxes.^5^

Numerous interventions have been shown to help smokers quit,^6^^,^^7^ but it is more difficult to detect changes in quit rates at the population level than in clinical trials.^8^ Annual smoking cessation rates were essentially unchanged from the early 1990s to 2010 despite the introduction of multiple quitting aids and a clinical practice guideline for treating tobacco use.^6^^,^^8^ Encouragingly, some studies have reported that population cessation rates increased in the second decade of the new millennium.^9^^,^^10^ The goal of cutting cancer deaths in half requires consideration of any interventions that could measurably increase population cessation.

Tobacco tax increases are considered one of the most effective interventions for reducing smoking prevalence.^11-13^ Raising taxes can discourage young people from initiating smoking and encourage current smokers to quit.^11-13^ It can also induce current smokers to decrease consumption.^14^^,^^15^ Moreover, raising taxes generates revenue that can potentially be used to fund tobacco prevention programs.^5^ From 2000 to 2022, all but 2 United States states raised their tobacco taxes, doing so nearly 3 times on average.^16^^,^^17^

In 2016, following years of efforts to increase California’s tax, advocates in the state promoted Proposition (Prop) 56, a voter initiative to raise the cigarette tax by $2.00 per pack, with equivalent increases on e-cigarettes and other tobacco products.^18^^,^^19^ Despite aggressive industry opposition,^20^^,^^21^ voters approved the initiative by a wide margin and the law took effect in April 2017.^19^ Revenue from the tax was used mostly to shore up California’s Medicaid program, but about 15% was earmarked for tobacco prevention spending.^18^

Studies conducted soon after the implementation of Prop 56 found that the tax increase was largely passed on to consumers.^22-24^ However, findings on the behavioral effects of the tax increase were mixed, due in part to the utilization of different datasets and analytical approaches. Gunadi et al.^23^ found that Prop 56 had no effects on smoking prevalence, cigarette consumption, or the prevalence of other tobacco product use. Boettiger and White, using a synthetic control, estimated that cigarette sales in 2018 were 16.6% lower than they would otherwise have been.^24^ Keeler et al.^25^ found decreased smoking prevalence among low-income adults, but not among high-income adults.

The present study focused on smoking cessation, aiming to determine whether Prop 56 was associated with a detectable increase in smoking cessation rates among adult smokers. Studies using regression analysis have generally found that increased price correlates with an increase in smoking cessation.^26-29^ The present study focused on a single state, California, which had a dramatic price increase due to Prop 56. Unlike earlier studies immediately following the implementation of Prop 56,^23-25^ the present study aggregated data for 3 years before and 3 years after implementation to provide more stable estimates of smoking cessation rates for these 2 periods. This study compared California with 2 groups of other states: those that also increased their tobacco taxes during the study timeframe, albeit to a lesser extent than California, and those that did not increase their taxes. The comparison with other states, especially those that had no tax increases, can offer insights into the effects of tobacco tax increases and on the future development of policies and programs to improve smoking cessation rates at the population level.

## Methods

### Data sources, sample, and study design

Smoking cessation data came from the Behavioral Risk Factor Surveillance System (BRFSS) for the years 2014-2019.^30^ BRFSS is a state-based, nationwide telephone survey on health-related risk behaviors conducted annually with state-representative samples of civilian, non-institutionalized people aged 18 years and older. For 2014-2019, the combined sample included 2 653 613 adults from all 50 states and the District of Columbia (DC). The study focused on ever smokers, that is, those who had smoked at least 100 cigarettes in their lifetimes. Within this population, it included only those who had smoked in the last year. The effective sample size was 443 054.

Data on state cigarette excise taxes were extracted from information published by the Federation of Tax Administrators.^16^ Data on cigarette retail prices by state were extracted from *The Tax Burden on Tobacco*.^31^ Data on state-level tobacco prevention spending were extracted from information published by the Campaign for Tobacco-Free Kids.^32^

The study design aggregated data into two 3-year periods roughly corresponding to before and after the implementation of Prop 56: 2014-2016 and 2017-2019, respectively. Because the initiative took effect in April 2017, the before and after periods were approximate. The 50 states and DC were categorized into 3 groups based on cigarette tax increases during the study period from 2014 to 2019: (1) California, (2) other states that raised their taxes during the study period (*n* = 18, including DC), and (3) states that did not raise their taxes (*n* = 32).

### Measures

The study focused on annual smoking cessation rates. Only respondents who had smoked cigarettes in the last 12 months were considered. Those who had quit smoking at the time of the survey were asked, “How long has it been since you last smoked a cigarette, even one or two puffs?”^30^ Those who had abstained for at least 3 months at that time were defined as 3-month former smokers and considered to have quit successfully. The main outcome measure was the annual population cessation rate, defined as the percentage of respondents who had smoked in the past 12 months who were 3-month former smokers at the time of the survey.^8^

A secondary measure was the percentage of current smokers who smoked daily at the time of the survey. This was used as a crude measure of dependence, as nondaily smokers are considered less dependent than daily smokers.^33^ Daily smoking is the only nicotine dependence measure in BRFSS.

### Statistical analysis

For each state, the average state cigarette tax and the average price of a cigarette pack were calculated separately for 2014-2016 and 2017-2019, along with the change (in dollars and percentage) between the 2 periods. State tobacco prevention spending data by fiscal year were used to estimate state spending by calendar year, which with census data were used to calculate average annual spending per capita for the 2 periods.^34^

Cigarette price and state spending data were adjusted for inflation. Prices were inclusive of taxes. Consumer Price Index (CPI) Historical Tables from the US Department of Labor were used for the inflation adjustment.^35^ We followed the method used in previous studies for computing monthly cigarette prices using CPI before aggregating the adjusted prices for each of the 3-year periods.^36^^,^^37^ All results are presented in 2019 dollars.

Established complex survey procedures from BRFSS were used in computing point estimates and 95% confidence intervals for cessation rates.^38^ Annual cessation data for 2014-2016 and for 2017-2019 were aggregated to obtain more stable estimates for each period. Because the BRFSS survey sample for each state varied by year (sometimes significantly), we used the Monte Carlo (parametric bootstrap) method to estimate the mean annual cessation rate for each state in each of the 3-year periods.^39^ In this approach, a Gaussian noise was added, consistent with the standard error for that state and year, to each year’s point estimate for each state. Then, we used linear regression to estimate the mean for each state in each 3-year period. We repeated this procedure 100 000 times and averaged the resultant means. Two-tailed z tests were used to compare these means.

All analyses were conducted in 2024 using SAS 9.4 software. The study used public datasets, and was approved by the Institutional Review Board at the University of California San Diego (#809580).

## Results

Table 1 shows, by state, average state taxes on a pack of cigarettes for the periods 2014-2016 and 2017-2019 and the magnitude of change between the 2 periods. All 50 states and DC assessed at least some tax, but the amounts varied widely. Taxes in 2017-2019 ranged from $0.17 in Missouri to $4.35 in New York. California and 18 other states (including DC) each raised their taxes at least once during the study period, whereas no states lowered them. Among those raising their taxes (marked with an asterisk in Table 1), the smallest increase was in Oregon, where the tax rose $0.01, or 1.0%. The largest increase was in California, where the tax rose $1.83, or 210.7%. (Note that Prop 56 raised the cigarette tax by $2.00, but because it took effect in April 2017, the average increase between the 2 periods was $1.83.)

**Table 1.** Average cigarette excise tax rates and cigarette prices by state, 2014-2016 and 2017-2019.

State	State excise tax per cigarette pack				Price per cigarette pack (CPI-adjusted)^a^			
	Average 2014-2016	Average 2017-2019	Change in	Change in	Average 2014-2016	Average 2017-2019	Change in	Change in
	$	$	$	%	$	$	$	%
Alabama*	0.53	0.68	0.15	27.6	5.57	5.66	0.09	1.7
Alaska	2.00	2.00	—	—	9.60	9.51	−0.09	−0.9
Arizona	2.00	2.00	—	—	7.33	7.40	0.06	0.9
Arkansas	1.15	1.15	—	—	6.03	6.15	0.12	2.0
California*	0.87	2.70	1.83	210.7	5.96	7.80	1.84	30.8
Colorado	0.84	0.84	—	—	5.85	5.92	0.07	1.2
Connecticut*	3.55	4.21	0.67	18.8	9.05	9.80	0.75	8.3
Delaware*	1.60	1.99	0.39	24.3	6.30	6.80	0.49	7.8
District of Columbia*	2.89	3.77	0.88	30.3	8.02	8.97	0.95	11.9
Florida	1.34	1.34	—	—	6.09	6.20	0.11	1.7
Georgia	0.37	0.37	—	—	5.16	5.24	0.09	1.7
Hawaii	3.20	3.20	—	—	9.56	9.42	−0.14	−1.5
Idaho	0.57	0.57	—	—	5.46	5.60	0.14	2.5
Illinois*	1.98	2.15	0.17	8.4	7.87	7.97	0.10	1.3
Indiana	1.00	1.00	—	—	5.82	5.83	0.01	0.2
Iowa	1.36	1.36	—	—	6.27	6.36	0.09	1.5
Kansas*	1.04	1.29	0.25	24.0	5.94	6.27	0.32	5.5
Kentucky*	0.60	0.85	0.25	41.7	5.23	5.56	0.32	6.1
Louisiana*	0.67	1.08	0.42	62.4	5.54	6.02	0.48	8.7
Maine	2.00	2.00	—	—	7.12	7.23	0.11	1.6
Maryland	2.00	2.00	—	—	7.03	7.09	0.06	0.8
Massachusetts	3.51	3.51	—	—	9.54	9.69	0.14	1.5
Michigan	2.00	2.00	—	—	7.09	6.94	−0.15	−2.2
Minnesota*	3.44	3.61	0.17	5.1	8.55	8.99	0.44	5.2
Mississippi	0.68	0.68	—	—	5.49	5.44	−0.04	−0.8
Missouri	0.17	0.17	—	—	4.88	5.02	0.14	2.9
Montana	1.70	1.70	—	—	6.78	6.79	0.01	0.2
Nebraska	0.64	0.64	—	—	5.69	5.67	−0.02	−0.4
Nevada*	1.30	1.80	0.50	38.5	6.28	6.73	0.45	7.1
New Hampshire	1.78	1.78	—	—	6.69	6.70	0.01	0.1
New Jersey	2.70	2.70	—	—	7.98	7.85	−0.12	−1.5
New Mexico*	1.66	1.72	0.06	3.4	6.86	6.82	−0.04	−0.6
New York	4.35	4.35	—	—	10.88	10.59	−0.29	−2.7
North Carolina	0.45	0.45	—	—	5.12	5.21	0.09	1.8
North Dakota	0.44	0.44	—	—	5.07	5.23	0.16	3.2
Ohio*	1.43	1.60	0.18	12.3	6.34	6.53	0.19	3.0
Oklahoma*	1.03	1.53	0.50	48.5	6.06	6.70	0.64	10.6
Oregon*	1.31	1.33	0.01	1.0	6.29	6.38	0.10	1.5
Pennsylvania*	1.74	2.60	0.86	49.5	7.10	8.22	1.12	15.7
Rhode Island*	3.62	4.15	0.53	14.6	9.10	9.64	0.54	5.9
South Carolina	0.57	0.57	—	—	5.37	5.38	0.01	0.2
South Dakota	1.53	1.53	—	—	6.53	6.64	0.11	1.7
Tennessee	0.62	0.62	—	—	5.40	5.40	—	—
Texas	1.41	1.41	—	—	6.32	6.42	0.10	1.6
Utah	1.70	1.70	—	—	6.86	6.92	0.07	1.0
Vermont*	2.89	3.08	0.19	6.5	8.47	8.68	0.21	2.4
Virginia	0.30	0.30	—	—	5.37	5.49	0.12	2.3
Washington	3.03	3.03	—	—	8.44	8.45	0.01	0.1
West Virginia*	0.66	1.20	0.54	82.3	5.39	6.10	0.71	13.2
Wisconsin	2.52	2.52	—	—	8.04	7.81	−0.23	−2.9
Wyoming	0.60	0.60	—	—	5.53	5.54	0.01	0.3

* indicates states that increased their cigarette excise taxes during the study period, 2014-2019.

Tax information based on Federation of Tax Administrators, Cigarette tax increases 2000-2022. 2023. Price data based on Orzechowski and Walker, *The Tax Burden on Tobacco, 1970–2019*. Arlington, VA: Orzechowski and Walker, 2020.

Abbreviation: CPI = consumer price index.

a Prices are in 2019 dollars.

Table 1 also shows, by state, average retail prices for a pack of cigarette for the 2 periods and the magnitude of change between the periods. Prices were adjusted for inflation, resulting in negative increases for some states. The smallest dollar increase in price was −$0.29 in New York, and the smallest percentage increase was −2.9% in Wisconsin. The largest increase was in California, where the price rose $1.84, or 30.8%.

Table 2 summarizes this information for California and 2 groups of other states. In states besides California that raised their taxes, average taxes rose $0.36 from 2014-2016 to 2017-2019, representing a 21.8% increase. In the same period, prices rose $0.43, or 6.3%. In states that did not raise their taxes, prices rose by only $0.01, or 0.2%.

**Table 2.** Average cigarette excise tax rates and cigarette prices in 3 groups of states, 2014-2016 and 2017-2019.

Group	State excise tax per cigarette pack				Price per cigarette pack (CPI-adjusted)^a^			
	Average 2014-2016	Average 2017-2019	Change in	Change in	Average 2014-2016	Average 2017-2019	Change in	Change in
	$	$	$	%	$	$	$	%
California	0.87	2.70	1.83	210.7	5.96	7.80	1.84	30.8
Other states with tax increases (*n* = 18)^b^	1.65	2.01	0.36	21.8	6.84	7.28	0.43	6.3
States with no tax increases (*n* = 32)^c^	1.71	1.71	—	—	6.88	6.89	0.01	0.2

States include the 50 US states and the District of Columbia. In grouping the states, only tax increases from 2014 to 2019 were considered. Tax information based on Federation of Tax Administrators, *Cigarette tax increases 2000–2022*. Price information based on Orzechowski and Walker, *The Tax Burden on Tobacco, 1970–2019*. Averages were weighted by state population.

Abbreviation: CPI = consumer price index.

a Prices are in 2019 dollars.

b Includes Alabama, Connecticut, Delaware, District of Columbia, Illinois, Kansas, Kentucky, Louisiana, Minnesota, Nevada, New Mexico, Ohio, Oklahoma, Oregon, Pennsylvania, Rhode Island, Vermont, and West Virginia.

c Includes Alaska, Arizona, Arkansas, Colorado, Florida, Georgia, Hawaii, Idaho, Indiana, Iowa, Maine, Maryland, Massachusetts, Michigan, Mississippi, Missouri, Montana, Nebraska, New Hampshire, New Jersey, New York, North Carolina, North Dakota, South Carolina, South Dakota, Tennessee, Texas, Utah, Virgina, Washington, Wisconsin, and Wyoming.

Table 3 shows average annual state tobacco prevention spending per capita, adjusted for inflation, for the same 3 groups of states. In California, spending per capita rose $4.94 (an increase of 271.9%) from 2014-2016 to 2017-2019. Among other states that raised their taxes, spending on average fell $0.21 (−12.8%). In states that did not raise their taxes, spending also fell, by $0.18 per capita (−11.2%).

**Table 3.** Average annual state-level per capita spending on tobacco prevention programs in 3 groups of states, 2014-2016 and 2017-2019.

Group	Annual per capita spending (CPI-adjusted)^a^			
	Average 2014-2016	Average 2017-2019	Change in	Change in
	$	$	$	%
California	1.82	6.75	4.94	271.9
Other states with tax increases (*n* = 18)	1.67	1.46	−0.21	−12.8
States with no tax increases (*n* = 32)	1.63	1.45	−0.18	−11.2

States include the 50 US states and the District of Columbia. In grouping states, only tax increases from 2014 to 2019 were considered. Based on Campaign for Tobacco-Free Kids, *Broken Promises to Our Children: A State-By-State Look at the 1998 Tobacco Settlement 24 Years Later*. Averages were weighted by state population.

Abbreviation: CPI = consumer price index.

a Prices are in 2019 dollars.

**Figure 1.**
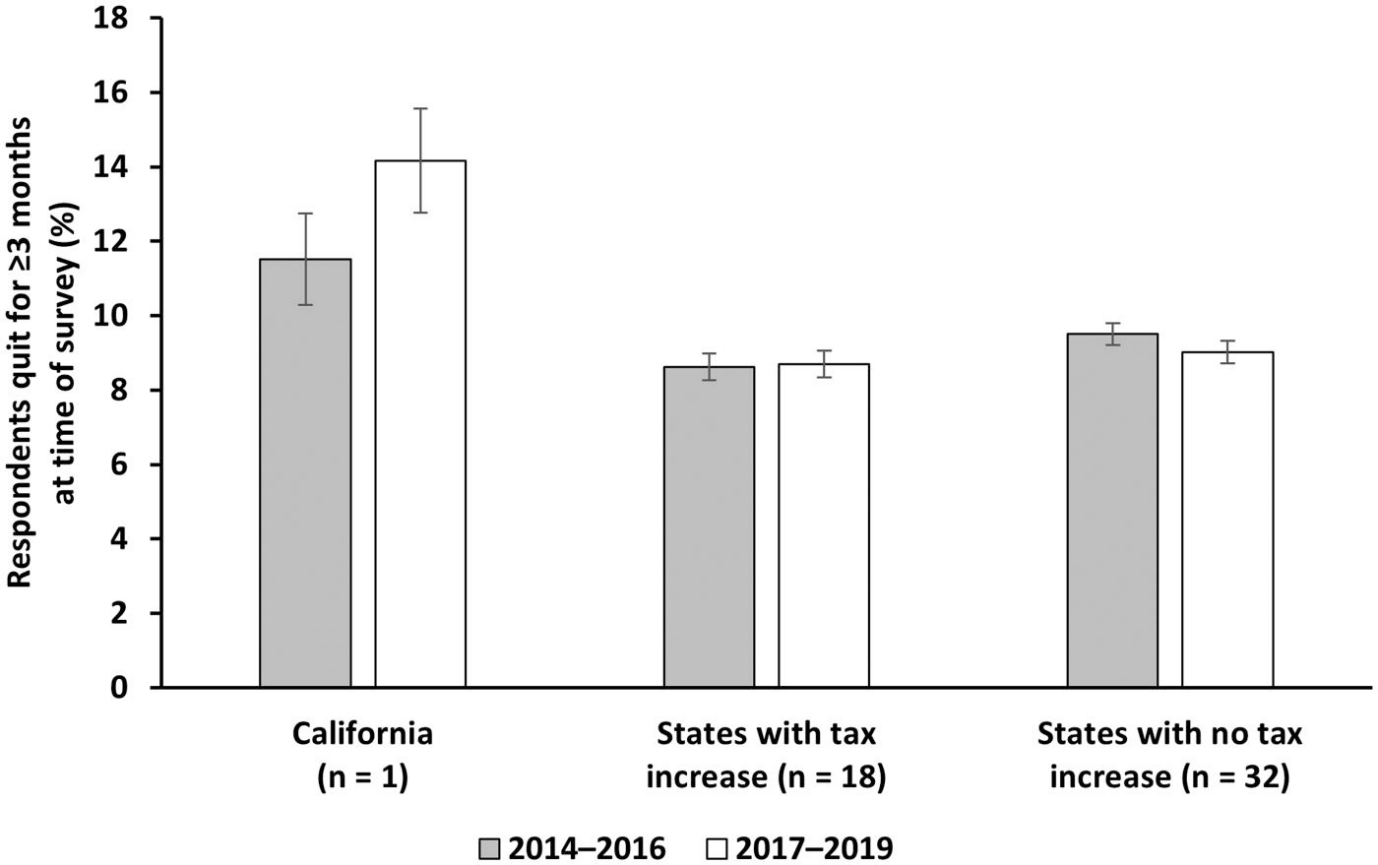
Smoking cessation rates among adult smokers for 3 groups of states in 2014-2016 and 2017-2019. States include the 50 US states and the District of Columbia. In grouping states, only tax increases from 2014 to 2019 were considered. Cessation rates based on data from the Behavioral Risk Factor Surveillance System, 2014-2019. Comparison between the 2 time periods for the 3 groups is as follows: California (*n* = 8698), difference = 2.6 percentage points, *P* = .005; 18 states with tax increase (*n* = 147 615), difference = 0.1 percentage points, *P* = .755; 32 states with no tax increase (*n* = 286 741), difference = −0.5 percentage points, *P* = .026.

Figure 1 shows annual smoking cessation rates for California and the 2 groups of other states. Rates increased significantly in California from 11.5% in 2014-2016 to 14.2% in 2017-2019 (*P* = .005). There was no significant change in cessation rates in other states that raised their taxes, at 8.6% and 8.7% for the 2 periods, respectively (*P* = .755). Cessation rates declined significantly in states that did not raise their taxes, from 9.5% to 9.0% (*P* = .026).

Table 4 shows the proportion of daily smokers among those who were still smoking at the time of the survey. In 2014-2016, this proportion was significantly lower in California (60.4%) than in other states that raised their taxes (71.2%) and in states that did not raise their taxes (68.4%). By 2017-2019, the proportion of daily smokers in California dropped 4.0 percentage points (*P* = .012). In contrast, there was no significant change in the proportion of daily smokers in the 2 groups of other states.

**Table 4.** Proportion of daily smokers among respondents who continued smoking in 3 groups of states, 2014-2016 and 2017-2019.

Group	2014-2016		2017-2019		Percentage point difference	*P*
	*n*	% (95% CI)	*n*	% (95% CI)		
California	3396	60.4 (58.3 to 62.5)	3270	56.4 (54.1 to 58.7)	−4.0	.012
Other states with tax increase (*n* = 18)	65 291	71.2 (70.5 to 71.8)	59 267	71.8 (71.2 to 72.4)	0.6	.164
States with no tax increase (*n* = 32)	125 411	68.4 (67.9 to 68.9)	116 313	68.3 (67.7 to 68.9)	−0.1	.879

States include the 50 US states and the District of Columbia. In grouping states, only tax increases from 2014 to 2019 were considered. Based on data from the Behavioral Risk Factor Surveillance System, 2014-2019.

Abbreviation: CI = confidence interval.

## Discussion

California’s $2-per-pack cigarette tax increase in 2017 was associated with a significant increase in population smoking cessation rates, from 11.5% in 2014-2016 to 14.2% in 2017-2019. Over the same timeframe, cessation rates did not change significantly in other states that raised their cigarette taxes and declined significantly in states that did not raise their taxes. Moreover, the proportion of daily smokers among those who did not quit smoking declined significantly in California, whereas for other states, the proportion of daily smokers remained essentially unchanged. Many factors likely contributed to the increased quitting in California; this study focused on 2 that could be directly measured in dollars: the tax increase itself and the associated increase in tobacco prevention spending that the tax increase enabled.

Before Prop 56, California’s cigarette tax ranked 35th in the United States, at $0.87 per pack. The steep increase in California’s tax from such a low starting point represented a proportional increase of over 200%. The attendant 30% hike in the retail price of cigarettes likely acted as a “jolt to the system,” prompting many smokers to quit.

The implementation of Prop 56 came with a substantial boost in tobacco prevention spending in California. State spending per capita in 2014-2016 was only $1.82, less than 20% of what the Centers for Disease Control and Prevention recommends.^5^^,^^32^ The initiative nearly quadrupled state spending per capita to $6.75 in 2017-2019.^5^^,^^32^ Meanwhile, in other states that raised their taxes, spending per capita actually declined 12.8%, demonstrating that tobacco tax increases do not always translate to increased spending on tobacco prevention.

Besides boosting quit rates, the tax increase and associated spending increase in California also appeared to reduce consumption among those who did not quit smoking. The proportion of daily smokers among those still smoking decreased significantly from 2014-2016 to 2017-2019, suggesting a possible “softening of the target” for future interventions, as less dependent smokers may be more likely to succeed in quitting smoking.^33^^,^^40^^,^^41^

In contrast, in states besides California that raised their tobacco taxes during the study period, no changes in smoking cessation rates were observed. This finding should be interpreted with caution because the before and after periods in this study were selected specifically to illuminate changes in quitting associated with California’s tax increase, using other states as comparison conditions. That said, the lack of change in these other states was unsurprising, given the difficulty of increasing cessation at the population level. For example, a review of national data from 1991 to 2010 found that annual population smoking cessation rates did not change significantly despite many efforts aimed at increasing quitting.^8^ One could argue that cessation rates in California during the study period might also have remained unchanged, had the state’s tax and spending increases been smaller.

The results for states that did not raise their taxes during the study period provide further support for this argument. At the beginning of the study, these states had taxes that on average were twice as high as California’s ($1.71 vs $0.87) because many had raised their taxes earlier. For example, New York had the highest tax, at $4.35 per pack; it did not raise its taxes further during the study period, nor was it surpassed by other states. In this group of states, the price of cigarettes rose only 0.2% from 2014-2016 to 2017-2019, whereas state tobacco prevention spending fell 11.2% and the cessation rate dropped significantly from 9.5% to 9.0%, representing nearly 150 000 fewer smokers quitting annually in these states. This shows that states in a relatively strong position with regard to taxation can nevertheless lose ground in the battle against tobacco. Realistically, there may be limits to how much or how often governments can raise tobacco taxes, suggesting that equal attention should be paid to shoring up tobacco prevention spending, such as by earmarking a greater share of tax revenues for this purpose.

This study has limitations. First, although Prop 56 was clearly associated with increased smoking cessation in California, it is difficult to determine how much the increase was due to the tax-induced price increase, to the attendant increase in tobacco prevention spending, or to other factors, such as social cues about the undesirability of smoking that smokers may have drawn from the overwhelming public support for the initiative. Second, the study used cross-sectional BRFSS survey data from multiple years but did not examine for clustering effects that could have occurred if the same individuals participated in multiple surveys, although that is unlikely to have been a problem. The study also did not examine cessation trends for the 3 groups of states before 2014, which could affect the estimates for the differences in cessation rates. Finally, the study did not fully address why initial smoking cessation rates were higher in California than in states that did not raise their taxes, which in turn were higher than in states that did raise their taxes. Table 4 provides some clues about this, as California had a lower baseline proportion of daily smokers than states that did not raise their taxes, which in turn had a lower baseline proportion than states that did raise their taxes. This is a complicated issue beyond the scope of the study to resolve.^42^ It is encouraging, however, that cessation rates increased even further in California after the implementation of Prop 56, despite the state’s higher initial rate of cessation.

Prop 56 provided an opportunity to obtain evidence for the effects of a tobacco tax increase on the population cessation rate in a single state, owing to California’s large population, the low starting point and much higher end point of its tax rates, and its re-investment of a substantial portion of new revenues in tobacco prevention, as well as the availability of concurrent data from other states for comparison. The results of this study have implications for governments considering raising tobacco taxes to accelerate cessation at the population level: to maximize impact, it would seem prudent to raise taxes by a substantial amount and to re-invest a significant portion of new revenues in tobacco prevention programming.

## Data Availability

Smoking cessation data in this paper are available from the Behavioral Risk Factor Surveillance System at https://www.cdc.gov/brfss/annual_data/annual_data.htm. State cigarette excise tax data are available from the Federation of Tax Administrators at https://taxadmin.memberclicks.net/cigarette-tax-increases. Cigarette price data are available from Orzechowski and Walker, *The Tax Burden on Tobacco, 1970–2019*, at https://healthdata.gov/dataset/The-Tax-Burden-on-Tobacco-1970-2019/etts-u9ii/data. The Consumer Price Index Historical Table is available from the Department of Labor Statistics at https://www.bls.gov/cpi/. Population weights are available from the US Census Bureau, American Community Survey, at https://data.census.gov/table?q=Hispanic&g=010XX00US$0400000. Data analysis scripts that generated results in this paper will be made available upon reasonable request.
